# A Carbon-Based Antifouling Nano-Biosensing Interface for Label-Free POCT of HbA1c

**DOI:** 10.3390/bios11040118

**Published:** 2021-04-12

**Authors:** Zhenhua Li, Jianyong Li, Yanzhi Dou, Lihua Wang, Shiping Song

**Affiliations:** 1 Division of Physical Biology, CAS Key Laboratory of Interfacial Physics and Technology, Shanghai Institute of Applied Physics, Chinese Academy of Sciences, Shanghai 201800, China; lzh@sinap.ac.cn (Z.L.); lijianyong18@mails.ucas.ac.cn (J.L.); douyanzhi@sinap.ac.cn (Y.D.); wanglihua@sinap.ac.cn (L.W.); 2 Zhangjiang Laboratory, The Interdisciplinary Research Center, Shanghai Synchrotron Radiation Facility, Shanghai Advanced Research Institute, Chinese Academy of Sciences, Shanghai 201210, China; 3 University of Chinese Academy of Sciences, Beijing 100049, China

**Keywords:** electrochemical biosensing, three-dimensional electron transporter, multi-walled carbon nanotubes, HbA1c, point-of-care testing

## Abstract

Electrochemical biosensing relies on electron transport on electrode surfaces. However, electrode inactivation and biofouling caused by a complex biological sample severely decrease the efficiency of electron transfer and the specificity of biosensing. Here, we designed a three-dimensional antifouling nano-biosensing interface to improve the efficiency of electron transfer by a layer of bovine serum albumin (BSA) and multi-walled carbon nanotubes (MWCNTs) cross-linked with glutaraldehyde (GA). The electrochemical properties of the BSA/MWCNTs/GA layer were investigated using both cyclic voltammetry and electrochemical impedance to demonstrate its high-efficiency antifouling nano-biosensing interface. The BSA/MWCNTs/GA layer kept 92% of the original signal in 1% BSA and 88% of that in unprocessed human serum after a 1-month exposure, respectively. Importantly, we functionalized the BSA/MWCNTs/GA layer with HbA1c antibody (anti-HbA1c) and 3-aminophenylboronic acid (APBA) for sensitive detection of glycated hemoglobin A (HbA1c). The label-free direct electrocatalytic oxidation of HbA1c was investigated by cyclic voltammetry (CV). The linear dynamic range of 2 to 15% of blood glycated hemoglobin A (HbA1c) in non-glycated hemoglobin (HbAo) was determined. The detection limit was 0.4%. This high degree of differentiation would facilitate a label-free POCT detection of HbA1c.

## 1. Introduction

Electrochemical biosensors hold great promise for the global healthcare industry owing to rapid, inexpensive, miniaturized analytical devices [1,2,3,4,5,6,7,8,9,10,11,12,13,14]. Nevertheless, it still remains challenging to develop electrochemical biosensors for practical point-of-care testing (POCT) systems. First, the matrix effect caused by other biomolecules than targets from human fluid interferes the target recognition process, delivering a high possibility of false positive results, so the biosensing interface should be highly antifouling. Second, the abundance of detected biomarkers in human fluid is much lower than that of irrelevant biomolecules, so the biosensor should have a high sensitivity and have a high differentiation ability. A typical application of such electrochemical biosensors is for the detection of blood glycated hemoglobin A (HbA1c).

HbA1c level detection has become a standard diagnostic method of diabetes control. The American Diabetes Association has recommended measuring HbA1c for diabetes screening and diagnosis [15]. In healthy adults, HbA1c is within the range of 4.0–6.0%, whereas levels >6.5% indicate diabetes [16]. Several kinds of electrochemical biosensing methods for HbA1c had been developed. Additionally, commercial tests are available. However, these commercial tests and devices are bulky and mainly used in clinical laboratory. Currently, POCT methods and devices are solely needed, but suffer from much more non-specific adsorption than laboratory tests because samples are often used without pretreatment in POCT [17]. Although a number of nanocomposite-based sensors have been developed for the detection of HbA1c [18,19,20,21,22], a high abundance of interfering components such as non-glycated hemoglobin (HbAo) or serum proteins in clinical matrices can greatly interfere with detection results. Thus, anti-biofouling must be considered [17,23,24].

To improve the detection sensitivity of electrochemical biosensors, a number of surface modification and biomolecular immobilization strategies were developed for the detection of nucleic acids [12,13,14,25], proteins [6,26], small molecules [27,28] and cells [10,29] in different sample matrixes. Typically, nanomaterials [30] with good conductivity such as gold nanoparticles [31,32], graphene [28] and carbon nanotubes [33,34], had been intensively applied to design electrochemical biosensing interfaces, facilitating the direct electron transfer between biomolecules and electrodes. In particular, CNTs can offer both a large surface-to-volume ratio and excellent electronic properties for biosensing analysis [30,35,36,37,38]. Nevertheless, the involvement of inorganic nanomaterials may absorb proteins and increase nonspecific adsorption [39,40]. To reduce the matrix effect during the electrochemical biosensing process, a common route is to decorate the electrode’s surface with highly biocompatible materials such as bovine serum albumin (BSA) and polyethylene glycol (PEG) [39,40,41,42,43]. However, the involvement of these materials would lead to the inevitable decrease in electrode conductivity, increasing the limit of detection (LOD) of electrochemical biosensors.

Recently, three-dimensional (3D) porous matrixes with antifouling properties have attracted much attention [44,45,46,47,48]. These strategies tried to combine the good conductivity of nanomaterials to the antifouling properties of 3D biological structures. For example, Jonathan et al. constructed an antifouling coating for electrodes consisting of a 3D porous matrix of cross-linked BSA supported by a network of conductive nanomaterials composed of gold nanowires and gold nanoparticles [44]. These nanocomposites allowed their electrochemical biosensor to operate in complex biological fluids such as blood plasma or serum. Nevertheless, such developed strategies suffer from two disadvantages. First, the need for noble metals would impede the industrial applications and the spread of these techniques. Second, complicated operations of these methods make them fail to meet the requirement of point-of-care diagnostics.

**Scheme 1:**
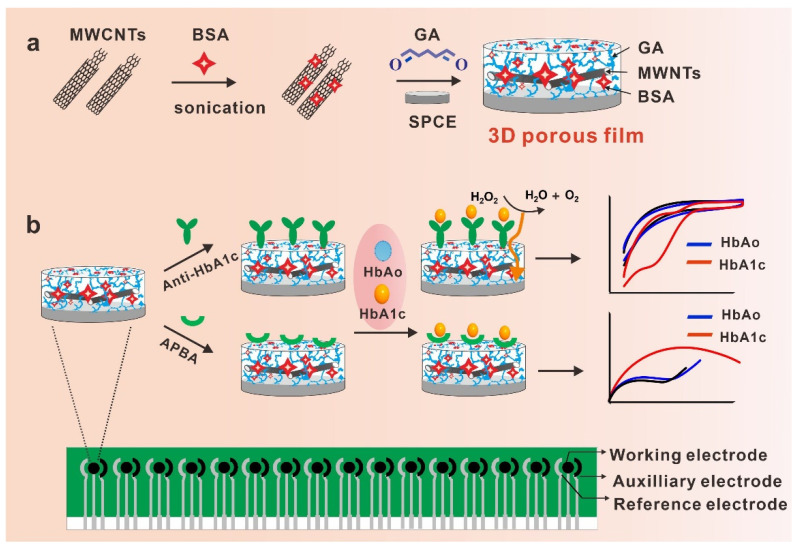
(**a**) Schematic illustration of the bovine serum albumin and multi-walled carbon nanotubes cross-linked with glutaraldehyde (BSA/MWCNTs/GA) layer on the surface of the screen-printed carbon electrodes (SPCE); (**b**) schematic illustration of the BSA/MWCNTs/GA-coated electrodes functionalized with different bioprobes for selective sensing of HbA1c.

Here, we designed a novel carbon-based nano-bio interface for electrochemical biosensors in combination of disposable screen-printed carbon electrodes (SPCE) with 3D nano-bio structures including BSA, multi-wall carbon nanotubes (MWNTs) and glutaraldehyde (GA). SPCE has many more advantages than other kinds of electrodes because of its low cost and feasibility of mass production [49,50,51,52,53,54,55]. We tried to functionalize a bovine serum albumin and multi-walled carbon nanotubes cross-linked with glutaraldehyde (BSA/MWCNTs/GA) layer with anti-HbA1c and 3-aminophenylboronic acid (APBA) for the detection of HbA1c and demonstrate the feasibility of the antifouling nano-interface for both large molecular probes and small molecular probes in the development of electrochemical biosensors (Scheme 1). Our results indicate that the BSA/MWCNTs/GA layer could both improve electrochemical performance and reduce non-specific binding, showing a great promise for developing simple, high-sensitivity and non-fouling biosensing platform to drive industrial application toward multi-scenario POCT.

## 2. Materials and Methods

### 2.1. Materials

BSA, hydrogen peroxide (H_2_O_2_), ethanolamine, 3-aminophenylboronic acid (APBA), carbodiimide hydrochloride (EDC), *N*-hydroxy-succinimide (NHS) and 70% GA were purchased from Sigma-Aldrich (St. Louis, MO, USA). HbAo, HbA1c, anti-HbA1c were purchased from Fitzgerld Industries International (Acton, MA, USA). MWNTs (50 nm in diameter, 1–2 μm in length) were purchased from Shenzhen Nanotech Port Co. Ltd. (Shenzhen, China). Normal human sera were obtained from Renji Hospital, School of Medicine, and Shanghai JiaoTong University. The 16-channel screen-printed carbon electrode (16-SPCE) was purchased from Zhejiang Nanosmart Biotechnical Co. Ltd. (Ningbo, China).

### 2.2. Fabrication of the Antifouling Layer

#### 2.2.1. The Preparation of MWCNT-Based Composites

In this process, 1.5 mg MWCNTs and 5.0 mg BSA were mixed in 1 mL 10 mM PBS to form the BSA/MWCNTs composite. Then, the mixture was sonicated in a sonicator (Q700, Qsonica) with a microtip for 30 min. After the sonication was done, the obtained solution was centrifuged for 15 min, the supernatant was recovered and ready for use.

#### 2.2.2. Electrode Layer Fabrication

The SPCE was pretreated electrochemically to clean the surface on their working electrode by running cyclic voltammetry (CV) with the 16-channel electrochemical detector. Then, 69 μL of BSA/MWCNTs composites in 10 mM PBS was directly mixed with 1 μL of 70% GA. The SPCE was kept in a 60% humidity box for 20 h at room temperature. After being rinsed twice with PBST buffer (10 mM phosphate, 140 mM NaCl, 2.7 mM KCl, 0.5% (*v*/*v*) Tween20, pH 7.4) and once with PBS (10 mM phosphate, 140 mM NaCl, 2.7 mM KCl, pH 7.4) in a shaker for 30 min, the BSA/MWCNTs/GA-modified SPCE can be stored in a N_2_ atmosphere at 4 °C for 4 weeks. The SPCE can also be functionalized by carbodiimide chemistry during this period. Independent SPCE arrays were used for all of the electrochemical experiments to produce statistical results.

### 2.3. BSA/MWCNTs/GA Layer Characterization and Optimization

#### 2.3.1. Characterization by UV Spectroscopy

The MWCNT-based composites were characterized by UV-vis absorption spectrophotometer (Hitachi U-3010, Tokyo, Japan) before and after addition of GA. The samples were diluted in 10 mM phosphate buffer.

#### 2.3.2. Characterization by SEM and AFM

The BSA/MWCNTs/GA layer was characterized using an atomic force microscope (AFM, Multimode Nanoscope VIII Instrument, Bruker, Billerica, MA, USA) and scanning electron microscope (SEM, LEO 1530 VP, Zeiss, Oberkochen, Germany). AFM images were obtained under tapping mode in air by using a RTESPA tip (Bruker).

#### 2.3.3. Electrochemical Measurements

All electrochemical experiments were carried out on 16-SPCE arrays. The nanocomposite layer were electrochemically characterized, using 5 mM K_4_Fe(CN)_6_/K_3_Fe(CN_)6_ prepared in 0.1 M KCl by CV (scan rate 100 mV/s between −0.2 and 0.6 V versus Ag/AgCl reference electrode) and electrochemical impedance spectroscopy (EIS) (0.1 MHz to 0.1 Hz, 5 mV amplitude versus Ag/AgCl reference electrode).

#### 2.3.4. BSA/MWCNTs/GA Layer Optimization and Antifouling Properties

The tested concentrations of MWCNTs were 0.5, 1, 1.5, and 2 mg/mL. Additionally, we chose the concentration of BSA (0, 0.1, 1 and 5 mg/mL) and the percentage of GA (0, 0.1, 1 and 5%) for the layer formation. The formation time of the layer was chosen (1 h, 4 h, 8 h, 18 h, 1 day and 2 days). Then, SPCEs were washed twice with PBST and once with PBS. The formed layer on SPCE was characterized electrochemically, using 5 mM K_4_Fe(CN)_6_/K_3_Fe(CN)_6_ prepared in 0.1M KCl by CV (scan rate 100 mV/s between −0.2 and 0.6 V versus Ag/AgCl reference electrode) at room temperature. For the antifouling experiment, the BSA/MWCNTs/GA layers were placed in 1% BSA and unprocessed human serum separately for 1 week, 2 weeks, 3 weeks and 1 month at 4 °C. Then, the layer on SPCE was characterized electrochemically, using 5 mM K_4_Fe(CN)_6_/K_3_Fe(CN_)6_ prepared in 0.1 M KCl by CV.

### 2.4. Biosensing of HbA1c Based on the Functionalized BSA/MWCNTs/GA Layer

#### 2.4.1. Functionalization of BSA/MWCNTs/GA Layer

The BSA/MWCNTs/GA layer was activated with 20 μL mixture solution of 0.05 M NHS in 10 mM PBS and 0.2 M EDC in 10 mM PBS for 30 min. Then, 20 μL of 25 μg/mL anti-HbA1c in 10 mM PBS or 10 mM APBA in 100 mM PB was added, following by incubation at 37 °C for 2 h. Then, 30 μL of 0.1 M ethanolamine in 100 mM PB was casted on the layer. Each step was washed twice with PBST and once with PBS.

#### 2.4.2. Selectivity Study of Functionalized BSA/MWCNTs/GA Layer

The anti-HbA1c- or APBA-functionalized BSA/MWCNTs/GA layer was incubated with 10 μL of 200 μg/mL HbAo and 6% HbAlc (containing 188 μg/mL of HbAo and 12 μg/mL of HbA1c) in unprocessed human serum at 37 °C for 5 min. After being washed twice with PBST and twice with PBS, the electrochemical analysis on anti-HbA1c-BSA/MWCNTs/GA layer was performed in 3 mM H_2_O_2_ containing 0.05 M PBS at room temperature by CV (scan rate 100 mV/s between −0.8 and 0 V). The electrochemical analysis on APBA-BSA/MWCNTs/GA layer was performed by EIS as described in Section 2.3.3.

#### 2.4.3. Biosensing of HbA1c Based on the Functionalized BSA/MWCNTs/GA Layer

HbA1c standards at variable concentrations (1, 2.5, 5, 10, 25 and 50 μg/mL) were prepared in unprocessed human serum. It is a consensus statement to use mmol mol^−1^ (IFCC) or % (NGSP) to report HbA1c concentrations. Here, we used the latter [56]. HbA1c at different percentile concentrations (2%, 4%, 6%, 9%, 12% and 15%) was prepared by serially diluting 200 μg/mL (100%) HbA1c with 200 μg/mL (0%) HbAo in unprocessed human serum. The electrochemical detection was measured using CV and EIS.

## 3. Results and Discussion

### 3.1. Design and Preparation of the BSA/MWCNTs/GA Layer

To decrease the LOD and antifouling properties of biosensors in clinical practice, here we designed an antifouling film for the sensing of HbA1c by the physical adsorption of BSA to multi-walled carbon nanotubes and then BSA cross-linking with GA to form a 3D porous matrix (BSA/MWCNTs/GA layer) onto SPCE. MWCNTs can offer both a large surface-to-volume ratio and excellent electric properties for biosensing analysis [35]. In our design, BSA is physically adsorbed to the surface of MWCNTs through hydrophobic interaction and then cross-linked with GA, producing 3D nanostructures in the form of porous film (that is, the BSA/MWCNTs/GA layer), as shown in Scheme 1a. The BSA/MWCNTs/GA layer not only displayed high resistance against nonspecific binding in complex biological fluids, but also exhibited excellent electron transfer ability. To evaluate the electrochemical sensing performance of the BSA/MWCNTs/GA layer, we functionalized the layer with anti-HbA1c antibodies and APBA for the detection of HbA1c in unprocessed human serum (Scheme 1b).

The consistency of the SPCE is one of the most important points to demonstrate the performance of the electrode. CV tests were carried out on 16-channel SPCE arrays. The relative standard deviation (RSD) of redox peak signals were evaluated as 2%, indicating a reliable repeatability (Figure S1a). To assess the electron transfer on BSA/MWCNTs/GA-coated electrodes, we also evaluated voltammograms at different scan rates. The currents were proportional to the square root of the scan rate, indicating a diffusion-limited process (Figure S1b,c).

In order to find the best composition of the BSA/MWCNTs/GA conjugate, the concentration of MWCNTs concentration was studied in the range of 0.5 to 2 mg/mL. The optimum concentration of MWCNTs was selected to be 1.5 mg/mL (Figure S2a). Then, BSA ranges from 0.1 to 5 mg/mL and GA from 0.1 to 5% were also investigated. The optimum composite 5 BSA/1 GA was chosen with the lowest drop in performance after 1-d incubation in 1% BSA (Figure S2b). Figure S2c showed that it required 18 h or more to obtain a stable BSA/MWCNTs/GA layer.

### 3.2. Structural Characterization of the BSA/MWCNTs/GA Layer and Its Performance

To confirm the formation of 3D porous structure, BSA/MWCNTs/GA was investigated by various methods. First, BSA/MWCNTs/GA was analyzed by measuring UV absorbance at 280 nm (Figure S3a). Compared to those without cross-linking through GA (BSA and BSA/MWCNTs), BSA/MWCNTs/GA and BSA/GA showed an increase in absorbance and the spectrum had shifted blue to 267–270 nm (Figure S3a), indicating that polymers of pyridine are yielded during BSA cross-linking by GA [57,58]. According to the rapid cross-linking mechanism, the produced polymers form 3D molecular networks. Then, atomic force microscopy (AFM) was used to further investigate and visualize the topography of BSA/MWCNTs/GA. As show in Figure S3b, the BSA-coated mica showed a very flat surface, while the topography of MWCNTs with the BSA can been seen on the BSA/MCWNT-coated mica. After cross-linking by GA, a sponge-like protein-MWCNT matrix was generated. Figure S3c shows the decrease in valleys from AFM results. These results prove that the cross-linking reaction of GA to the BSA/MWCNTs composite increased the roughness of the layer due to pore formation. Additionally, similar results were observed by scanning electron micrography (SEM) (Figure S3d).

**Figure 1:**
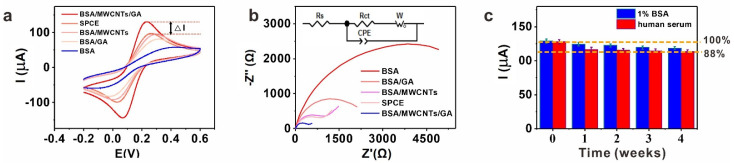
(**a**) The cyclic voltammetry (CV) characterization of different modified SPCEs. ΔI% = (I_BSA/MWCNTs/GA_ − I_SPCE_)/I_SPCE_; (n = 3, only one CV plot for each condition is shown); (**b**) the electrochemical impedance spectroscopy (EIS) characterization of different modified SPCEs. (n = 3, only one EIS plot for each condition is shown) Inset: Equivalent circuit of impedance measurement; (**c**) the stability of BSA/MWCNTs/GA layer in 1% BSA and unprocessed human serum. (n = 3, error bars represent the standard deviation of the mean).

To demonstrate the electrochemical properties of the BSA/MWCNTs/GA layer, the bare, BSA-, BSA/GA-, BSA/MWCNTs- and BSA/MWCNTs/GA-modified SPCE were prepared and investigated in 5 mM K_4_Fe(CN)_6_/K_3_Fe(CN_)6_ by CV and EIS. As shown in Figure 1a, the bare SPCE displayed a pair of quasi-reversible redox peaks, while the BSA-coated SPCE displayed redox peak signals reduced by 57% than those observed for the bare SPCE. The result should be attributed to the electrode passivated by non-conductive BSA. The modification of BSA/GA, BSA/MWCNTs on SPCE resulted in increased current responses than that of BSA, indicating that the presence of GA and MWCNTs accelerated the electron transport process. Significantly, the current response based on BSA/MWCNTs/GA modification was a 32% increase over the bare SPCE, demonstrating that the layer could provide a rapid and reliable path for electron transfer.

The impedance data can be fitted to a Randles circuit by using elements including constant phase element (CPE), charge transfer resistance (Rct), warburg element (W) and electrolyte solution resistance (Rs). In this work, Zview2 impedance software was used to fit the experimental data to the equivalent circuit to obtain Rct, which was used for further evaluation. Electrochemical responses of differently modified SPCE were also tested by EIS with curves shown in Figure 1b. The BSA layer exhibited the biggest resistance (6138 Ω), suggesting that it hindered the electron transfer. On the BSA/GA layer, the Rct value declined to 2426 Ω, indicating that the presence of GA-cross-linked BSA accelerated the electron transport process, and the Rct of the BSA/MWCNTs layer further declined to 1270 Ω due to conductivity of MWCNTs. Importantly, the Rct of the BSA/MWCNTs/GA layer declined to 469 Ω, proving that the nano-integrated layer could reduce the interface resistance and accelerate the electron transfer significantly.

It is crucial to demonstrate the performance of biosensors in complex biological fluids. Thus, we challenged the electrochemical biosensors with the BSA/MWCNTs/GA layer. The modified SPCEs were incubated in 1% BSA and unprocessed human serum separately. Impressively, the BSA/MWCNTs/GA layer showed excellent stability in these fluids during a 4-week period, keeping the original signal of 92% in 1% BSA and 88% in unprocessed human serum after one month, respectively (Figure 1c).

### 3.3. Biosensing of HbA1c Based on the Functionalized BSA/MWCNTs/GA Layer

The measurement of HbA1c level has become the standard of diabetes control. Here, the catalytic reduction of H_2_O_2_ by HbA1c on the BSA/WCNTs/GA layer was investigated to evaluate its electrochemical biosensing performance. Figure S4 shows the impedance of anti-HbA1c immobilization and then ethanolamine block. After the immobilization of anti-HbA1c, impedance values were significantly increased, compared with the impedance of the BSA/WCNTs/GA layer. Then, the experimental parameters for the electrocatalytic response of H_2_O_2_ by HbA1_C_ were optimized. As shown in Figure S5a, the peak current increased with increasing concentration until 3.0 mM H_2_O_2_, above which the values did not steeply increase. Therefore, 3.0 mM H_2_O_2_ was optimal. Figure S5b showed the effect of temperature from 20 to 40 °C. The peak current increased gradually and then decreased at 40 °C. This tendency might be due to the thermal deactivation of HbA1c. For practical applications, the subsequent experiments were performed at 37 °C. Figure S5c showed the effect of pH value. At pH 7.4, the current response was the maximum and used as the optimal pH value. Figure S5d showed the effect of applied potential. The response current increased and reached the steady state over at −0.60 V. The application of potential more than −0.60 V did not contribute more to the current response. Hence, the potential of −0.60V was used for the subsequent experiments.

**Figure 2:**
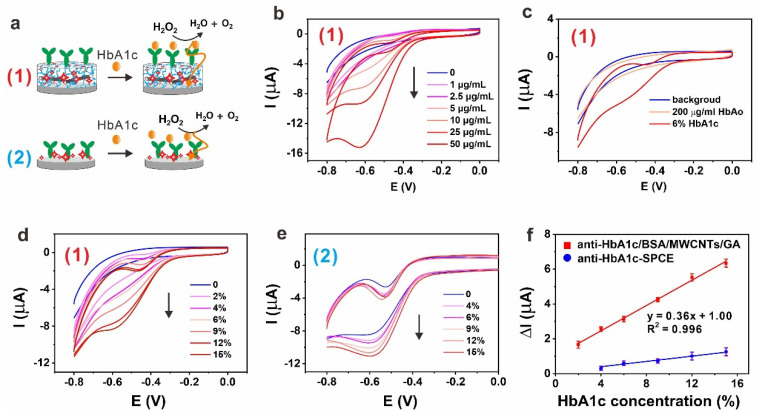
(**a**) Schematic illustration of anti-Hb1Ac-BSA/MWCNTs/GA layer (1) and anti-HbA1c layer (2) for selective sensing of HbA1c; (**b**) CV curves of the anti-HbA1c-BSA/MWCNTs/GA layer in the detection of different concentrations of HbA1c (no HbAo). (n = 3, only one CV plot for each condition is shown); (**c**) CV curve of the anti-HbA1c-BSA/MWCNTs/GA layer in the detection of HbAo and 6% HbA1c. (n = 3, only one CV plot for each condition is shown); (**d**) CV curves of the anti-HbA1c-BSA/MWCNTs/GA layer in the detection of different concentrations of % HbA1c (contain HbAo). (n = 3, only one CV plot for each condition is shown); (**e**) CV curves of the anti-HbA1c layer in the detection of different concentrations of % HbA1c (contain HbAo). (n = 3, only one CV plot for each condition is shown); (**f**) calibration curves as a function of % HbA1c for anti-HbA1c layer and anti-HbA1c-BSA/MWCNTs/GA layer. (n = 3, error bars represent the standard deviation of the mean).

The principle of HbA1c sensing on the anti-HbA1c-BSA/MWCNTs/GA layer is illustrated in Figure 2a(1) (anti-HbA1c modified bare SPCE as a contrast, Figure 2a(2)). HbA1c solution was serially diluted and detected by the electrochemical biosensor. The reduction current of H_2_O_2_ increased with the increase in HbA1c concentration (Figure 2b). The value of redox peak was proportional to the amount of HbA1c in the sample. The electrocatalytic response of H_2_O_2_ for the HbA1c detection was linear from 1 to 50 μg/mL (Figure S6). A regression equation (ΔI_P_(μA) = 0.24[HbA1c] + 0.69, R^2^ = 0.995) was obtained from the linear fitting. The limit of detection (LOD) of HbA1c was 0.6 μg/mL, which was calculated from 3σ/slope, where σ is the standard error of blank. This result shows that the BSA/MWCNTs/GA layer can detect HbA1c with low LOD.

A high abundance of non-glycated hemoglobin (HbAo) or serum proteins can interfere with the biosensor’s performance. Thus, we investigated the selectivity for HbA1c in relation to HbAo. Figure 2c shows CV plots for assaying 200 μg/mL HbAo and 6% HbA1c in unprocessed human serum. Upon the introduction of HbAo, CV plots virtually remained unchanged in comparison with the redox peak of the anti-HbA1c modified SPCE in the absence of HbAo. Thus, no redox peaks were observed. When the 6% HbA1c was captured on the anti-HbA1c-BSA/MWCNTs/GA layer, a catalytic reduction peak of H_2_O_2_ appeared at −0.5 V. This result shows that the biosensor can distinguish HbA1c from HbAo clearly, demonstrating its availability for accurate measurement of HbA1c levels of diabetic individuals.

Figure 2d shows CV plots acquired after incubation with different % HbA1c concentrations for the anti-HbA1c-BSA/MWCNTs/GA layer—the peak current increased with increasing concentration of % HbA1c, and the concentration differentiation is obviously better than that of anti-HbA1c layer (Figure 2e). These results indicate that the anti-HbA1c-BSA/MWCNTs/GA layer could efficiently prevent non-specific protein adsorption as compared to pure anti-HbA1c layer. A regression equation (ΔI_P_(μA) = 0.36 [HbA1c](%) + 1.00, R^2^ = 0.996) was obtained from linear fitting. The LOD of HbA1c was 0.4%, which was calculated from 3σ/slope (Figure 2f).

**Figure 3:**
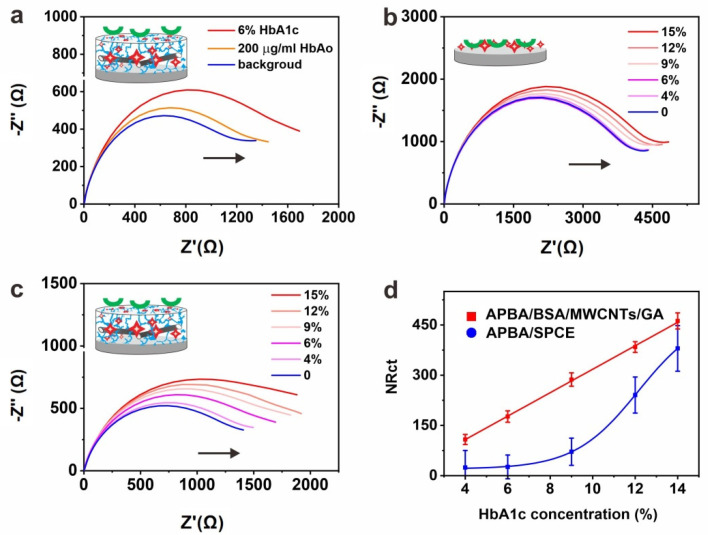
(**a**) EIS response of the APBA-BSA/MWCNTs/GA layer in the detection of HbAo and 6% HbA1c. (n = 3, only one EIs plot for each condition is shown); (**b**) EIS curve of the APBA layer in the detection of % HbA1c (contain HbAo). (n = 3, only one EIS plot for each condition is shown); (**c**) EIS curve of the APBA-BSA/MWCNTs/GA layer in detection of % HbA1c (contain HbAo). (n = 3, only one EIs plot for each condition is shown); (**d**) calibration curves as a function of % HbA1c for APBA layer and APBA-BSA/MWCNTs/GA layer. (n = 3, error bars represent the standard deviation of the mean).

Having demonstrated that the BSA/MWCNTs/GA layer can benefit the immobilization of large bioprobes such as antibodies of HbA1c, we also tried to demonstrate the availability of the layer for small bioprobes. Thus, we used APBA to functionalize the BSA/MWCNTs/GA-based SPCE for the detection of HbA1c by EIS. Figure 3a shows the selectivity of the APBA-based biosensor for HbA1c detection. An increase in the membrane resistance was observed in the presence of increasing HbA1c concentrations (Figure 3b,c). The normalized Rct was plotted vs. HbA1c concentration (Figure 3d), demonstrating that the response was linear from 4.0 to 15% of HbA1c in in unprocessed human serum with a LOD of 1.2% (3σ/slope). The EIS detection results demonstrate the versatility of the BSA/MWCNTs/GA layer for both large and small bioprobes in the development of HbA1c biosensors.

## 4. Conclusions

A three-dimensional nano-integrated BSA/MWCNTs/GA layer was developed to improve both the efficiency of electron transfer and the antifouling ability of biosensing surfaces. The BSA/MWCNTs/GA layer could keep 92% and 88% of the original signal in 1% BSA and unprocessed human serum after a 1-month exposure, respectively. The label-free direct electrocatalytic oxidation of HbA1c was explored by cyclic voltammetry (CV) on the BSA/MWCNTs/GA layer-based biosensor. The dynamic range of HbA1c in HbAo was determined from 2 to 15% with a LOD of 0.4%. Significantly, selective lab-free EIS detection demonstrated the versatility of the BSA/MWCNTs/GA layer for general electrochemical biosensing applications. We believe that such a nano-integrated biosensing strategy holds great potential for the development of POCT devices.

## Data Availability

Not applicable.

## Supplementary Materials

The following are available online at https://www.mdpi.com/article/10.3390/bios11040118/s1, Figure S1: (a) CV curves of 16-channel SPCE array; (b) CV curves of BSA/MWCNTs/GA layer of an equimolar solution of 5 mM ferri-/ferrocyanide at different scan rates 50–550 mV s^−1^; (c) Extracted oxidation/reduction peak current (i_p_) mean values from the CV shown in b plotted versus the square root of the scan rate, Figure S2: Optimization of the preparation of BSA/MWCNTs/GA layer in terms of (a), MWCNTs concentration; (b), BSA and GA ratio; (c), incubation time, Figure S3: (a) UV-Vis absorption spectra of BSA, BSA/MWCNTs, BSA/GA and BSA/MWCNTs/GA; (b) AFM topographies of micas coated with BSA, BSA/MWCNTs and BSA/MWCNTs/GA; (c) Line profile based on BSA, BSA/MWCNTs and BSA/MWCNTs/GA coated micas; (d) SEM images of SPCEs coated with BSA, BSA/MWCNTs and BSA/MWCNTs/GA, Figure S4: EIS curve of immobilization anti-HbA1c and APBA on BSA/MWCNTs/GA layer, Figure S5: Optimization of the experiment parameters in terms of (a) H_2_O_2_ concentration; (b) pH; (c) Applied potential for catalytic H_2_O_2_ reduction; (d) Temperature, Figure S6: Calibration curves for detection of HbA1c for anti-HbA1c-BSA/MWCNTs/GA layer.
