# Supplementary material for: A Carbon-Based Antifouling Nano-Biosensing Interface for Label-Free POCT of HbA1c
Source: Biosensors (Basel). 2021 Apr 12;11(4):118. doi: 10.3390/bios11040118 (PMC8069255; doi:10.3390/bios11040118)
Supplement: Supplementary file 1 [file biosensors-11-00118-s001.pdf]

Supplementary Information

A Carbon-Based Antifouling Nano-Biosensing Interface for Label-Free POCT of HbA1c

Zhenhua Li[1,2], Jianyong Li[1,2], YanzhiDou[1,2], Lihua Wang[3], Shiping Song[3]*

[1]Division of Physical Biology, CAS Key Laboratory of Interfacial Physics and Technology, Shanghai Institute of Applied Physics, Chinese Academy of Sciences, Shanghai 201800, China
[2]University of Chinese Academy of Sciences, Beijing 100049, China
[3]The Interdisciplinary Research Center, Shanghai Synchrotron Radiation Facility, Zhangjiang Laboratory, Shanghai Advanced Research Institute, Chinese Academy of Sciences, Shanghai 201210, China

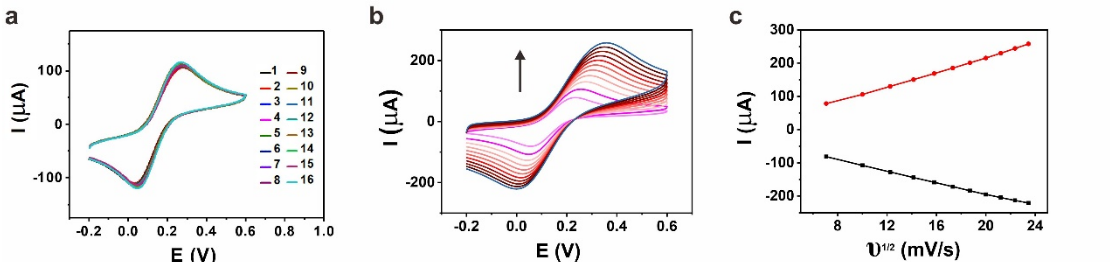

Figure S1. a, CV curves of 16-channel SPCE array. The number 1 to 16 represent 16 electrodes on the SPCE. **b,** CV curves of BSA/MWCNTs/GA layer of an equimolar solution of 5 mM ferri-/ferrocyanide at different scan rates 50-550 mV s^{-1}. **c,** extracted oxidation/reduction peak current (i_p) mean values from the CV shown in b plotted versus the square root of the scan rate. (n = 3 independent electrodes).

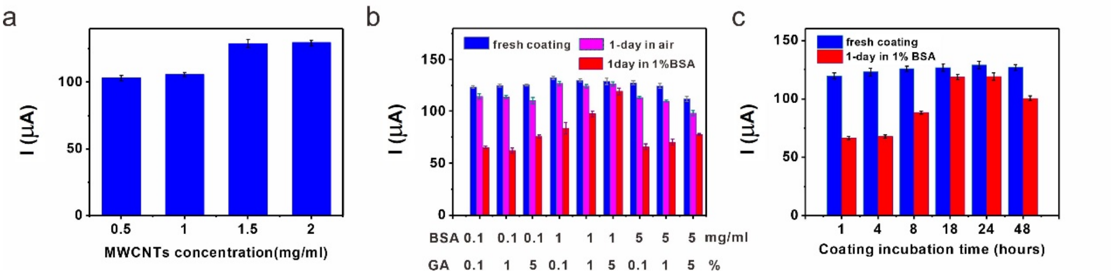

Figure S2. Optimization of the preparation of BSA/MWCNTs/GA layer in terms of **a**, MWCNTs concentration. **b**, BSA and GA ratio. **c**, incubation time. (n = 3, error bars represent the standard deviation of the mean).

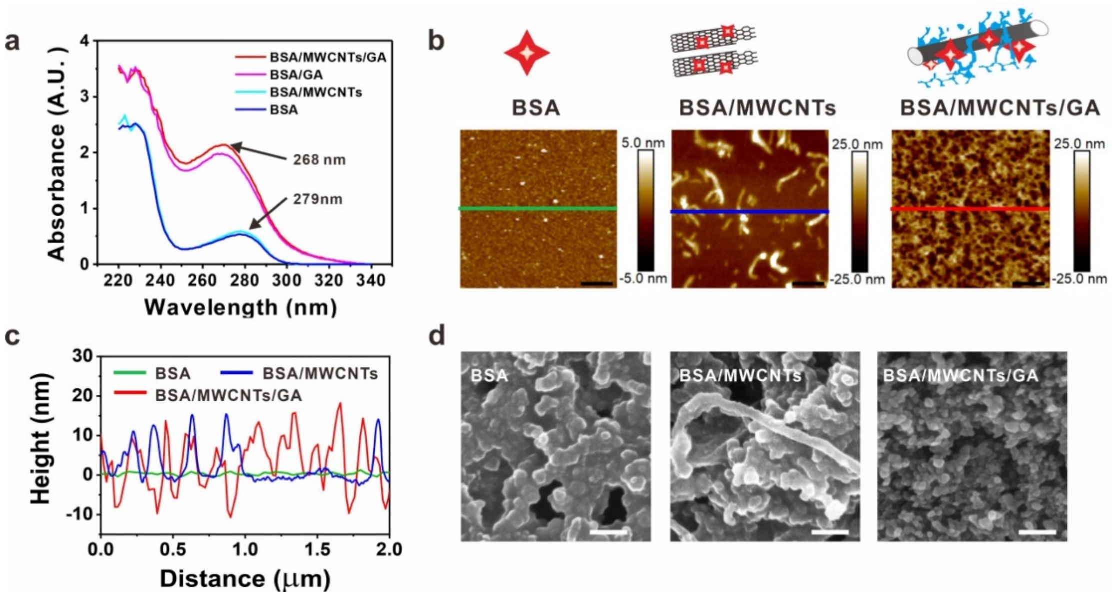

Figure S3. (**a**) UV-Vis absorption spectra of BSA, BSA/MWCNTs, BSA/GA and BSA/MWCNTs/GA (n = 3, only one UV-Vis absorption spectrum for each condition is shown). (**b**) AFM topographies of micas coated with BSA, BSA/MWCNTs and BSA/MWCNTs/GA (scale bar: 400 nm). This analysis was repeated twice on different samples. (**c**) Line profile based on BSA, BSA/MWCNTs and BSA/MWCNTs/GA coated micas. (**d**) SEM images of SPCEs coated with BSA, BSA/MWCNTs and BSA/MWCNTs /GA (scale bar: 100 nm). This analysis was repeated twice on different samples.

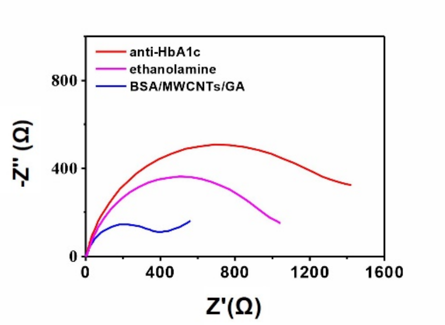

Figure S4. EIS curve of immobilization anti-HbA1c on BSA/MWCNTs/GA layer. (n = 3 independent electrodes, only one Nyquist plot for each condition is shown).

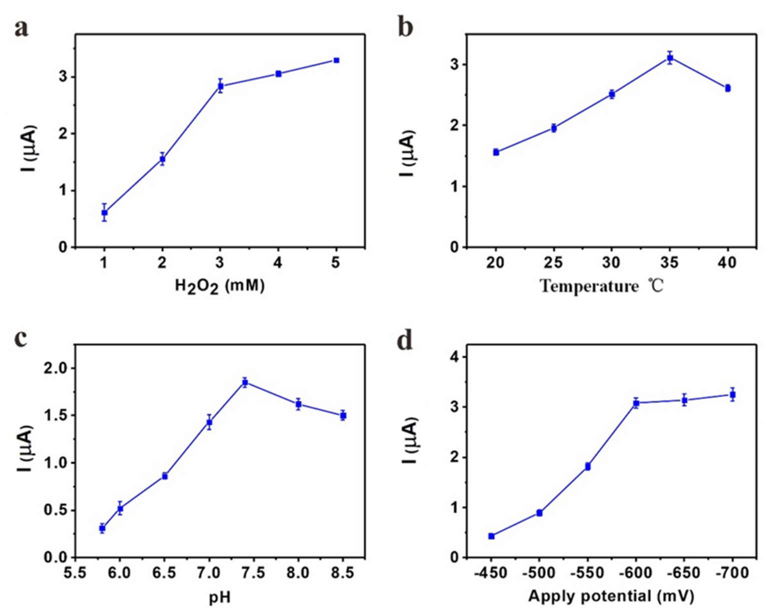

Figure S5. Optimization of the experiment parameters in terms of **a**, H_2O_2 concentration. **b**, temperature. **c**, pH. **d**, applied potential for catalytic H_2O_2 reduction. (n = 3, error bars represent the standard deviation of the mean).

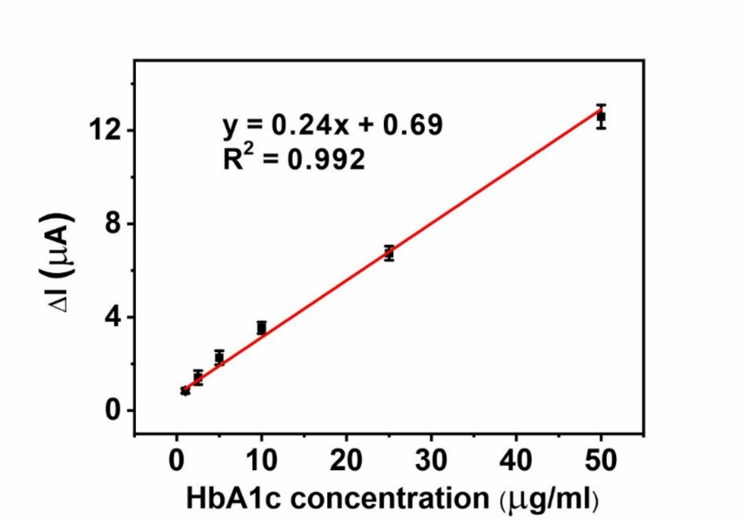

Figure S6. Calibration curves for detection of HbA1c for anti-HbA1c-BSA/MWCNTs/GA layer. (n = 3, error bars represent the standard deviation of the mean).
